# Mitophagy-mediated molecular subtypes depict the hallmarks of the tumour metabolism and guide precision chemotherapy in pancreatic adenocarcinoma

**DOI:** 10.3389/fcell.2022.901207

**Published:** 2022-07-22

**Authors:** Hao Chen, Jianlin Zhang, Xuehu Sun, Yao Wang, Yeben Qian

**Affiliations:** ^1^ Department of General Surgery, The First Affiliated Hospital of Anhui Medical University, Hefei, China; ^2^ Department of Emergency Surgery, The First Affiliated Hospital of Anhui Medical University, Hefei, China

**Keywords:** pancreatic adenocarcinoma, mitophagy, metabolism, ICGC data portal, the cancer genome atlas program

## Abstract

**Background:** Mitophagy is closely related to cancer initiation and progression. However, heterogeneity with reference to mitophagy remains unexplored in pancreatic adenocarcinoma (PAAD).

**Materials and methods:** We used Reactome database to download the mitophagy-related, glycolysis-related and cholesterol biosynthesis-related signaling pathways. Unsupervised clustering using the “ConsensusClusterPlus” R package was performed to identify molecular subtypes related to mitophagy and metabolism. Prognosis-related mitophagy regulators were identified by univariate Cox regression analysis. Receiver operating characteristics (ROC) and Kaplan-Meier (K-M) survival analyses were used to assess the diagnostic and prognostic role of the hub genes and prognosis risk model. Weighted gene co-expression network analysis (WGCNA) was utilized for screening the mitophagy subtype-related hub genes. Metascape was utilized to carry out functional enrichment analysis. The “glmnet” R package was utilised for LASSO, and the “e1071” R package was utilised for SVM. Chemotherapeutic drug sensitivity was estimated using the R package “pRRophetic” and Genomics of Drug Sensitivity in Cancer (GDSC) database. The nomogram was established by the “rms” R package.

**Results:** Three distinct mitophagy subtypes (low, high and intermediate) of PAAD were identified based on the landscape of mitophagy regulators. The high mitophagy subtype had the worst prognosis, highest mRNA expression-based stemness index scores and most hypoxic environment compared to the other subtypes. Additionally, glycolysis and cholesterol biosynthesis were significantly elevated. Three mitophagy subtype-specific gene signatures (*CAST, CCDC6,* and *ERLIN1*) were extracted using WGCNA and machine learning. Moreover, PAAD tumours were insensitive to Erlotinib, Sunitinib and Imatinib in the high mitophagy subtype and high *CAST, CCDC6,* and *ERLIN1* expressed subtypes. Furthermore, *CAST, CCDC6,* and *ERLIN1* affected immune cell infiltration (M1 and CD8Tcm), resulting in the altered prognosis of patients with PAAD. A nomogram was constructed to screen patients with the low mitophagy subtype, which showed a higher sensitivity to chemotherapeutic agents.

**Conclusion:** Based on various bioinformatics tools and databases, the PAAD heterogeneity regarding mitophagy was systematically examined. Three different PAAD subtypes having different outcomes, metabolism patterns and chemosensitivity were observed. Moreover, three novel biomarkers that are closely associated with mitophagy and have the potential to guide individualised treatment regimens in PAAD were obtained.

## Introduction

Pancreatic Adenocarcinoma (PAAD) is the deadliest form of digestive system cancer, with a 5-years survival rate of less than 10%. PAAD is an aggressive disease and is often diagnosed at an advanced stage when effective treatment options are lacking (Ding et al., 2018). Moreover, PAAD is projected to emerge as the second leading cause of cancer-related death by 2030 (Siegel et al., 20182018). The treatment-refractory nature of PAAD and limited clinically-validated biomarkers capable of predicting treatment response hinder the efficacy of PAAD therapeutics (Zeng et al., 2019). PAAD is heterogeneous cancer with distinguishable molecular subtypes and characteristics. Exploring the molecular subtypes at the transcriptome level can greatly contribute to the identification of clinically relevant biomarker signatures and prognostic strata (Collisson et al., 2019). These findings can aid in the personalisation of treatment regimens and the development of novel therapeutics.

Mitophagy is the cellular degradation of the damaged mitochondria *via* the mechanism of autophagy (Supplementary Figure S1) (Yoo and Jung, 2018). The inactivation of mitophagy leads to the accumulation of dysfunctional mitochondria in tumours. Studies have reported increased autophagy or mitophagy levels in various cancer types (Bernardini and LazarouM. Dewson, 2017). Compared to non-tumour cells, the majority of pancreatic tumour cells demonstrate highly fragmented mitochondria, which is closely related to increased mitochondrial fission and mitochondrial oxidative phosphorylation or glycolysis (Xie et al., 2021). Therefore, exploring the heterogeneity of mitochondrial biogenesis and turnover is important for the development of next-generation PAAD treatments.

One of the most investigated signaling pathways in mitophagy was PINK1/Parkin pathway. The serine/threonine PINK1 is the pivotal factor of the PINK1/Parkin pathway. Usually, the Translocase of the Outer Membrane and Translocase of the Inner Membrane (TIM) complexes transport PINK1 to the inner mitochondrial membrane (IMM) (Rasool et al., 2022). Subsequently, the presenilin-associated rhomboid-like (PARL) cleaves and degrades PINK1 on the IMM to maintain low PINK1 levels. Mitochondrial depolarization, resulting from the damaged mitochondria, prevents PINK1 translocation, which results in PINK1 phosphorylation by pyruvate dehydrogenase kinase isozyme 2 (PDK2) (Shi and McQuibban, 2017). This leads to PINK1 accumulation at the outer mitochondrial membrane (OMM) and Parkin, a U3 ubiquitin ligase, recruitment. PINK1 phosphorylates the serine 65 of Parkin’s ubiquitin-like domain, promoting its E3 ubiquitin ligase activity. Meanwhile, as key mitochondrial proteins, MFN1, MFN2, VDAC1, and Miro1 are ubiquitinated by Parkin to clear the damaged mitochondria (Glauser et al., 2011) (Chen and Dorn, 2013). Additionally, PINK1 phosphorylates these ubiquitin chains and activates Parkin, which leads to the amplification of mitochondrial phagocytosis signaling. The polyubiquitylation of the mitochondrial proteins leads to the interaction of the protein fragments with LC3 to form a complex, which is mediated by p62 and OPTN. This complex is subsequently degraded by the autophagic machinery (Wong and Holzbaur, 2014) (Geisler et al., 2010).

Generally, mitophagy suppresses PAAD tumour cell growth at the initial stage by limiting DNA damage or inflammation, whereas active mitophagy in the advanced stage promotes tumour cell survival by limiting cell death or anti-tumour immunity (Xie et al., 2021). Furthermore, mitophagy affects the occurrence and development of PAAD *via* tumour metabolic reprogramming (Ferro et al., 2020). For example, mitophagy induces lipid degradation and fatty acid oxidation, which provides materials for ATP production and thereby promotes PAAD growth. Moreover, Parkin deficiency increases mitochondrial dysfunctions, which leads to increased ROS production and glycolysis (Panigrahi et al., 2020). This contributes to the Warburg effect and consequently increases tumorigenesis.

Therefore, mitophagy holds great potential in the development of PAAD therapeutics. Furthermore, exploring the tumour heterogeneity of mitophagy will significantly contributes toward PAAD prevention and treatment.

## Materials and methods

### Data acquisition and processing

The clinical information, somatic mutation data and transcriptomic profiling of the discovery cohort was downloaded in The Cancer Genome Atlas (TCGA)-PAAD (Tomczak et al., 2015). In TCGA-PAAD, the 182 tissue samples were obtained from 176 patients with PAAD. Moreover, the transcriptome data of normal pancreatic tissue was obtained from the Genotype-Tissue Expression (GTEx) database (GTEx Consortium, 2013). The TCGA-PAAD and GTEx cohorts were merged to create a larger cohort (TCGA & GTEx) with 171 normal and 179 tumour samples.

Additionally, validation cohorts were acquired from the International Cancer Genome Consortium (ICGC) database and Gene Expression Ominbus (GEO) database, including the PACA-AU cohort, GSE60980, GSE71729 and GSE74629 (ICGC/TCGA Pan-C ancer Analysis of Whole Genomes Consortium, 2020) (Barrett et al., 2013). Detailed clinical information and transcriptomic profiles of the validation cohorts were also downloaded.

Signaling pathway search for mitophagy was retrieved from Reactome database using the key words: “mitophagy.” Three pathways (R-HSA-5205647, R-HSA-5205685, R-HSA-8934903) and 28 mitophagy regulators were obtain (Jassal et al., 2020). Additionally, Supplementary Table S1 also presented the information of cholesterol biosynthesis (R-HSA-191273) and glycolysis (R-HSA-70171) signaling pathways acquired from the Reactome database.

### Identification of mitophagy subtypes

Unsupervised hierarchical clustering using the R package “ConsensusClusterPlus” was performed to identify the expression patterns of mitophagy regulators (Wilkerson and Hayes, 2010). Mitophagy subtypes were acquired by the following parameter: reps = 50, pItem = 0.8, pFeature = 1, and distance = Euclidean. By performing unsupervised hierarchical clustering with the same parameters for all genes obtain from cholesterol biosynthesis and glycolysis pathways, different metabolic subtypes in the TCGA-PAAD cohort were obtained.

### Co-expression network construction

Co-expression networks were constructed using the weighted gene co-expression network analysis (WGCNA) R package (Langfelder and Horvath, 2008). The transcriptional profiles of the 9,221 differentially expressed genes between PAAD and normal tissues were downloaded from the GEPIA database (|Log2FC| > 1; FDR < 0.01) and used as input files for WGCNA (Tang et al., 2017). Median Absolute Deviation (MAD) was computed for each gene and the 50% of genes having lower MAD were eliminated. The “goodSamplesGenes” function in the WGCNA R package was utilised to remove outlier samples and genes. Subsequently, We performed WGCNA to establish a scale-free co-expression network based on the transcriptional profiles after screening. Initially, we used average linkage method to establish Pearson’s correlation matrices for all pair-wise genes. Subsequently, we utilized a power function to build a weighted adjacency matrix. The power function was as follows:

$$A_{\mathrm{ab}}=\left|C_{\mathrm{ab}}\right|\wedge \beta$$

(C_ab_ = Pearson’s correlation between gene a and gene b; A_ab_ = adjacency between Gene a and Gene b).

As a soft-thresholding power, the primary role of *β* was to emphasize strong correlations between the genes and penalize weak correlations. The topological overlap matrix (TOM) was transformed from the adjacency after we chosed the *β* of 12.

On selecting the power of 12, the adjacency was transformed into a topological overlap matrix (TOM) (Shuai et al., 2021). We utilized average linkage hierarchical clustering to partition genes with similar expression profiles into gene modules after setting the criterion as the a minimum size (gene group) of 30 and a sensitivity of 3. Additionally, the modules were merged if the distance between them was equal to or less than 0.25, which resulted in 11 modules.

Two methods were utilised to distinguish the modules closely linked with the clinical features of interest. Module Eigengene (ME) was evaluated by calculating the first principal component of each gene module. MEs represented the expression patterns of all the genes in module (Weiss et al., 2012). Correlations were computed between MEs and clinical features to obtain the gene module most related to the mitophagy subtypes. Furthermore, we performed linear regression between gene expression and clinical features and calculated the *p-*value of each gene (lg*p*) as gene significance (GS). Subsequently, module significance (MS) was obtained by calculating the average GS of all genes in the respective module. MSs were estimated to incorporate mitophagy subtypes of interest into the co-expression network.

### Pathway and process enrichment analyses

We utilized Metascape web tool to carry out pathway enrichment analyses (Zhou et al., 2019). Terms were deemed to be significantly enriched by a *p*-value < 0.01, minimum count of 3 and an enrichment factor >1.5. Then, Metascape web tool estimated the membership similarities between enriched items and classified them to diferent clusters. We evaluated *p*-value accroding to the accumulative hypergeometric distribution, and used Benjamini–Hochberg procedure to obtain *q*-value. In hierarchical clustering ,we set the similarity metric as kappa scores = 4, and items with similarity of >0.3 were recognized as one cluster. We next showed the enriched terms withe with smallest *p* values as the representative for each cluster.

### Drug sensitivity prediction

Drug-response prediction was evaluated using the “pRRophetic” package in R. Furthermore, Ridge regression was performed using the “pRRophetic” algorithm, which calculated the half-maximal inhibitory concentration (IC50) of each patient (Geeleher et al., 2014). Internal cross validation was carried out with ten-fold cross validation. The Genomics of Drug Sensitivity in Cancer (GDSC) database was used for the above calculations (Yang et al., 2013).

### Machine learning for the candidate mitophagy subtype-specific gene signature

In TCGA-PAAD cohort, the least absolute shrinkage and selection operator COX (LASSO-COX) and the support vector machines-recursive feature elimination (SVM-RFE) algorithms in R package “glmnet” and “e1071,” respectively, were used to screen candidate MSGSs (Engebretsen and Bohlin, 2019) (Xu et al., 2021). LASSO-COX compressed insignificant coefficients to zero *via* the penalized function. Therefore, this approach reduced the dimension of the feature space vector.

As a backward sequence selection algorithm, SVM-RFE stems from the maximum interval principle of SVM. The ten-fold cross-validation algorithm was used as the resampling method for SVM-RFE. The average importance of each feature in each iteration was considered as the final importance of features (Sanz et al., 2018).

The intersection of the results between the two methods was used for further analysis.

### Analysis of single cell RNA-seq data

Single-cell RNA-seq data of two untreated patients with PAAD from GSE111672 was selected for further analysis. The single cell expression profile matrix for GSE111672 was downloaded from the GEO database. Cell annotation in GSE111672 was performed using the Tumour Immune Single-cell Hub (TISCH) database (Sun et al., 2021). A total of 11 cell types were identified, including acinar, CD8Tcm, ductal, endothelial, fibroblasts, M1, malignant, monocyte, tprolif and neutrophils. According to the median levels of MSGSs, samples in GSE111672 were divided into two groups, which were dependent on the high or low expression level of MSGSs. PAAD tissues consist of a mixture of tumour cells and non-malignant cells; therefore, the proportion of intratumoral non-malignant cells was compared across different groups.

### Tumour-infiltrating immune cell analysis

Based on the RNA-seq data of the TCGA-PAAD cohort, the Immune Cell Abundance Identifier (ImmuCellAI) web tool was employed to describe the abundance of 24 immune cell types, including 18 T-cell subtypes and 6 other immune cells: B cell, NK cell, monocyte cell, macrophage cell, neutrophil cell and DC cell (Miao et al., 2020).

ImmuCellAI collected the specific gene sets from previous studies as gene signatures and acquired the reference expression profile from the GEO database for each cell type. Subsequently, ImmuCellAI evaluated the total expression deviation of all signatures in the input gene expression data matrix compared with the reference expression profiles of the 24 immune cell types. Single sample gene set enrichment analysis was performed to calculate the enrichment score of the gene signature to the corresponding immune cell type. Moreover, a compensation matrix was introduced and the weight of the shared genes on those immune cells was estimated using least square regression to re-assess their abundance.

ImmuCellAI analysis can be performed on either RNA-Seq or microarray expression data.

### Protein levels of MSGSs in the human protein atlas database

The HPA was designed to describe all human proteins in normal or tumour tissues through the integration of various omics technologies. The data of MSGSs in normal and PAAD tissues at the protein level were obtained from the HPA database (Song, Du, Gui, Zhou, Zhong, Mao, et al.).

### Statistical analyses

R software (R version 4.0.4) was used for statistical analyses. *p*-values were calculated by the nonparametric Wilcoxon test, which compared between two groups, while the Kruskal–Wallis test was used for multiple comparisons. The chi-square test was utilized to examine categorical variables. Kaplan–Meier survival analysis for overall survival (OS) and progression-free survival (PFS) was performed between different subgroups, followed by the log-rank test (Rich et al., 2010). A receiver operating characteristic (ROC) curve was constructed to assess the predictive efficacy of the prognostic prediction model (Mandrekar, 2010). A *p*-value less than 0.05 was considered statistically significant.

## Results

### Landscape and diagnostic role of mitophagy regulators in pancreatic adenocarcinoma

**FIGURE 1:**
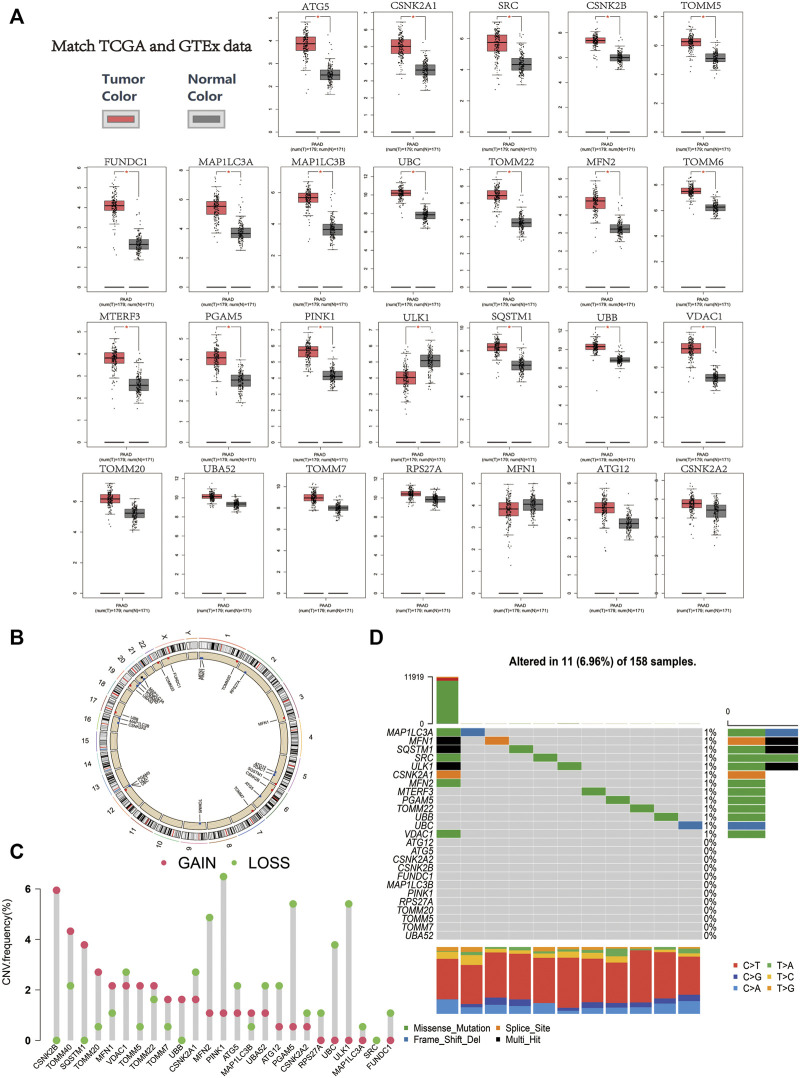
**(A)** Relative expression level of the mitophagy regulators in the PAAD samples in comparison to the expression of normal pancreatic tissue (red color represents tumor tissue, and black color represents normal tissue; red asterisk represents a *p*-value ≤ 0.01). **(B)** Chromosomal location of mitophagy regulators. **(C)** The CNV variation frequency of mitophagy regulators in TCGA cohort. The height of the column represented the alteration frequency (red indicates copy number gain, whereas green indicates copy number loss). **(D)** Waterfall (oncoplot) plot of the mitophagy regulators in TCGA cohort.

The RNA expression data of 179 PAAD samples and 171 normal samples from TCGA and GTEx databases were analysed using GEPIA. Limma method with FDR <0.01 and |log2FC| > 1 were chosen as the screening criteria for gene differential analysis. Notably, 19 mitophagy regulators were upregulated and one downregulated (ULK1) in PAAD (Figure 1A).

The position of the mitophagy regulators and their copy number variation regions are presented in Figure 1B. In the TCGA-PAAD cohort, CSNK2B and TOMM40 showed significantly higher CNC gain, whereas PINK1 and ULK1 showed significantly higher CNV loss (Figure 1C). In addition, the mutations of the mitophagy regulators were uncommon in the TCGA-PAAD cohort (6.96%; Figure 1D).

**FIGURE 2:**
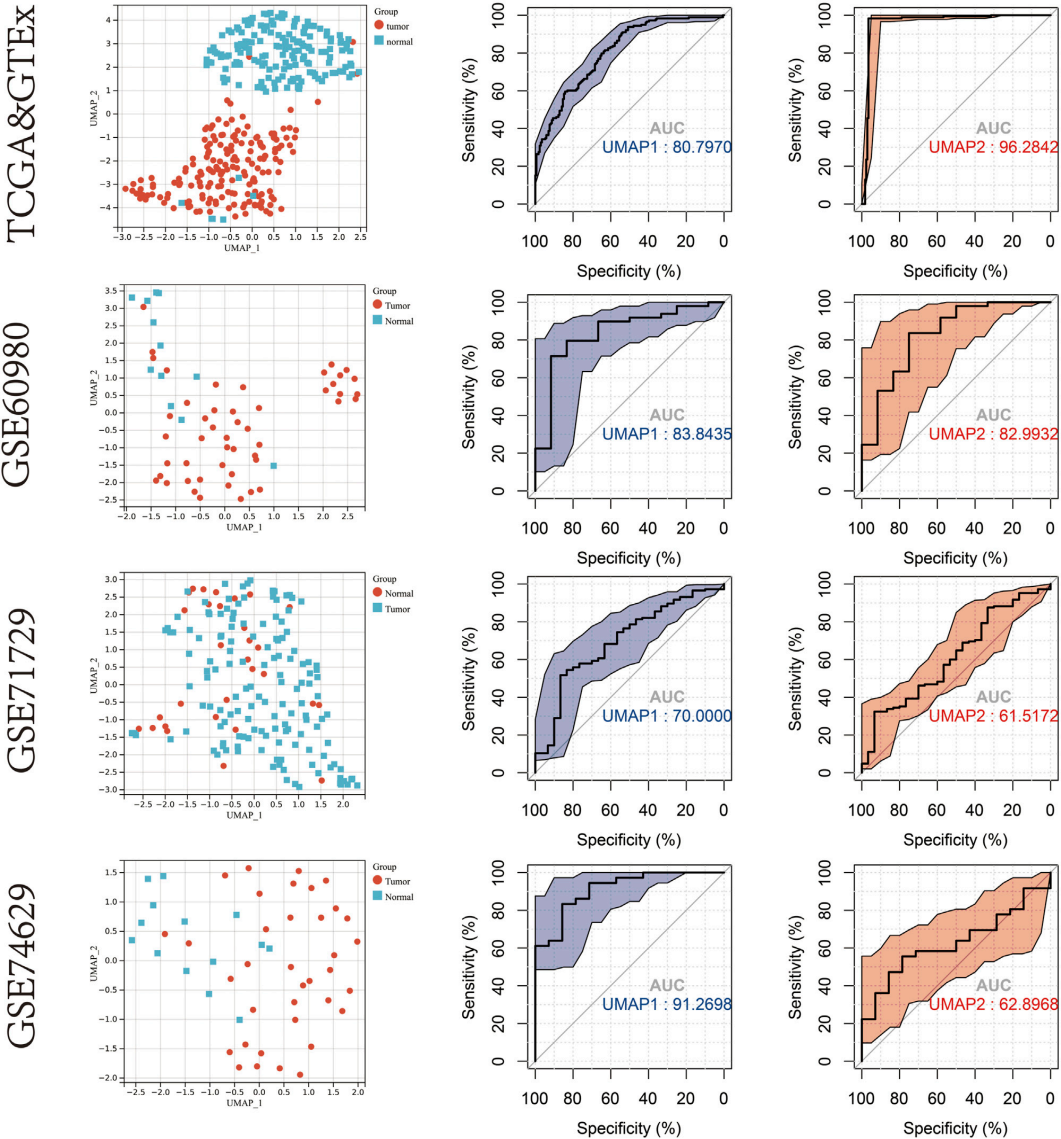
UMAP cluster representation of different mitophagy regulators expression patterns. ROC plot for diagnostic accuracy of UMAP1 and UMAP2 in TCGA & GTEx cohort,GSE60980,GSE71729 and GSE74629.

Uniform manifold approximation and projection (UMAP) dimensionality reduction was performed using the R package “umap” (version 0.2.7.0) on the transcriptome profiles of the 19 mitophagy regulators that were differently expressed between PAAD tumours and normal samples. Gene expression profiles were reduced to two-dimensional space (UMAP1 and UMAP2) for visualisation. The diagnostic value of UMAP1, UMAP2 in identifying PAAD and non-tumour samples were determined using ROC curve analysis (Figure 2). In the TCGA & GTEx cohort, the area under the curve (AUC) of the ROC curve for UMAP1 and UMAP2 was 0.808 and 0.963, respectively. Further verification of the diagnostic abilities of the mitophagy regulators on PAAD using UMAP analysis in the GSE60980, GSE71729 and GSE74629 datasets showed similar results.

### Construction of an mitophagy-related prognostic model for pancreatic adenocarcinoma patients

Excluding patients who had less than 3 months of follow-up in the TCGA-PAAD cohort (n = 10), the prognostic value of mitophagy regulators in terms of OS was evaluated using univariate COX analysis (Supplementary Figure S2A; Supplementary Table S2). Among the all mitophagy regulators, three genes were risk factors (*SRC, VDAC1,* and *MFN1*) and two were protective factors (*MAP1LC3A* and *ULK1*) for PAAD prognosis. Based on the expression profiles of the above 5 prognosis-related mitophagy regulators, LASSO analysis further identified four genes (*SRC, MFN1, MAP1LC3A* and *ULK1*), which were ultimately used in the construction of the prognostic model (Supplementary Figure S2B). The formula for the prognostic model was:

Riskscore = (-0.297∗MAP1LC3A exp.) + (-0.232∗ULK1 exp.) + (0.219∗SRC exp.) + (0.075∗VDAC1 exp.)

Based on this formula, the risk score of each patient in the TCGA-PAAD cohort was computed and ranked. The patients were classified into the high-risk or low-risk group according to the median value. The K–M curve indicated a high level of risk score suggesting a poor prognosis (Log rank *p-*value = 0.0035; Supplementary Figure S2C). The ROC curve for 5-years OS indicated an outstanding predictive value (AUC: 0.86) of this prognostic model (Supplementary Figure S2D).

### Description of mitophagy subtypes in pancreatic adenocarcino

**FIGURE 3:**
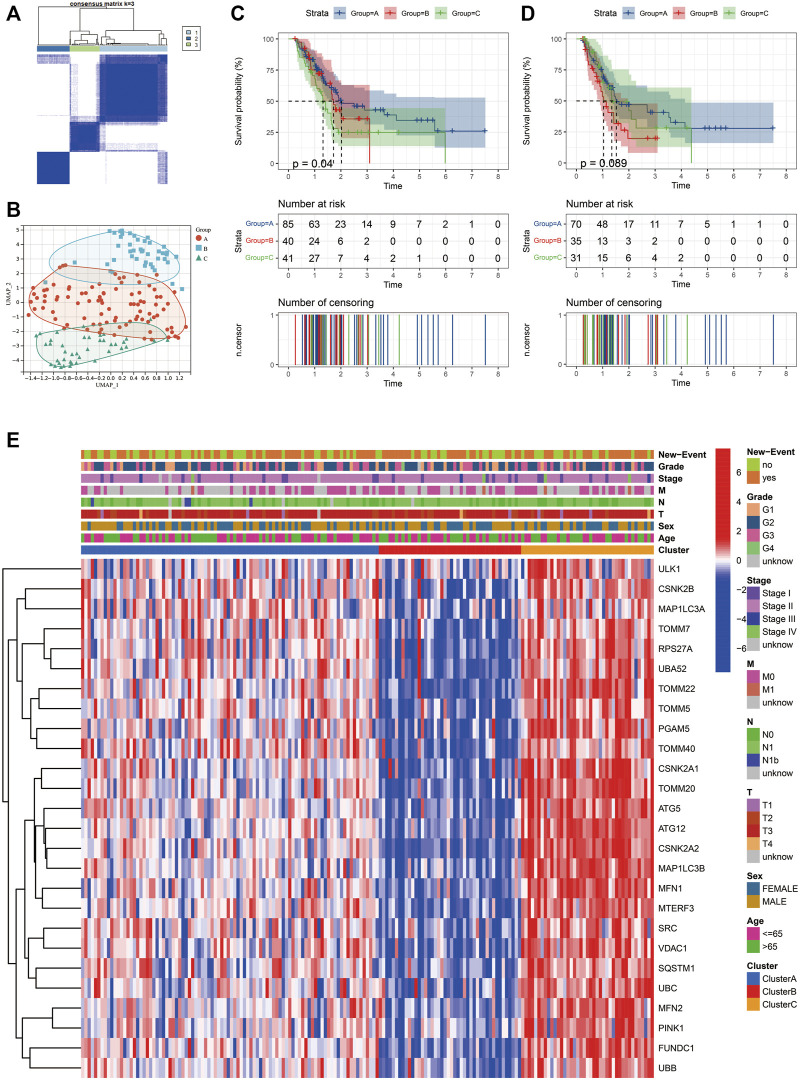
Identify different mitophagy subtypes in TCGA-PAAD. **(A)** Consensus matrices of the TCGA-PAAD cohort for *k* = 3. **(B)** Complete transcriptome profiles of mitophagy regulators are reduced to UMAP1 and 2 for visualization in TCGA-PAAD cohort. K-M analyses of OS **(C)** and PFS **(D)** for different mitophagy subtypes in TCGA-PAAD. **(E)** Heat map comparing expression levels of mitophagy regulators among the 3 mitophagy subtypes.

This study used unsupervised clustering to classify PAAD into three diverse molecular subtypes (cluster A, cluster B and cluster C) based on the mitophagy regulators *via* the R package “ConsensusClusterPlus” (Figure 3A). Twenty-six mitophagy regulators were included into unsupervised clustering analysis after excluding the regulators that could not be detected in the TCGA-PAAD cohort or whose expression levels were zero in more than half of the samples. We observed marked differences in the transcriptional profiles of the mitophagy regulators among the three different molecular subtypes (Figure 3B).

Furthermore, the OS and PFS of PAAD patients were analysed performing the K–M survival curves. Survival analyses indicated that the median survival of cluster A was 2.02 years; cluster B, 1.66 years; cluster C, 1.30 years. Moreover, patients in cluster C had a significantly worst prognosis than the other subtypes (log rank test; *p* = 0.04; Figure 3C). Additionally, patients in cluster B had the shortest PFS (Figure 3D ). Heatmap of the three mitophagy subtypes of PAAD using the 26 mitophagy regulators revealed distinct gene expression patterns (Figure 3E). Stage, age, gender and new events were utilized as patient annotations. Chi-square tests indicated that stage, age, gender, and new events did not differ significantly in the three subtypes. The highest gene expression levels of most mitophagy regulators appeared in cluster C, followed by cluster A and then cluster B (Supplementary Figure S3A). Therefore, the samples were denoted as low mitophagy (cluster B), high mitophagy (cluster C) and intermediate mitophagy (cluster A) subtypes.

Unsupervised clustering analysis was also performed in the validation cohort (ICGC-PACA-AU) following the same workflow. Similarly, samples in the validation cohort cluster were separated into three clusters (Supplementary Figure S3B). A significant difference was observed in prognosis among the three clusters (Supplementary Figure S3C). The expression patterns of the 26 mitophagy regulators were similar to that of the TCGA-PAAD cohort (Supplementary Figure S3D).

Characteristics of different Mitophagy Subtypes: Hypoxia, cancer stem cells (CSCs) and Metabolic Alterations.

To evaluate the difference in prognosis in the TCGA-PAAD cohort, hypoxia level, CSCs and metabolic patterns were compared among the three mitophagy subtypes.

**FIGURE 4:**
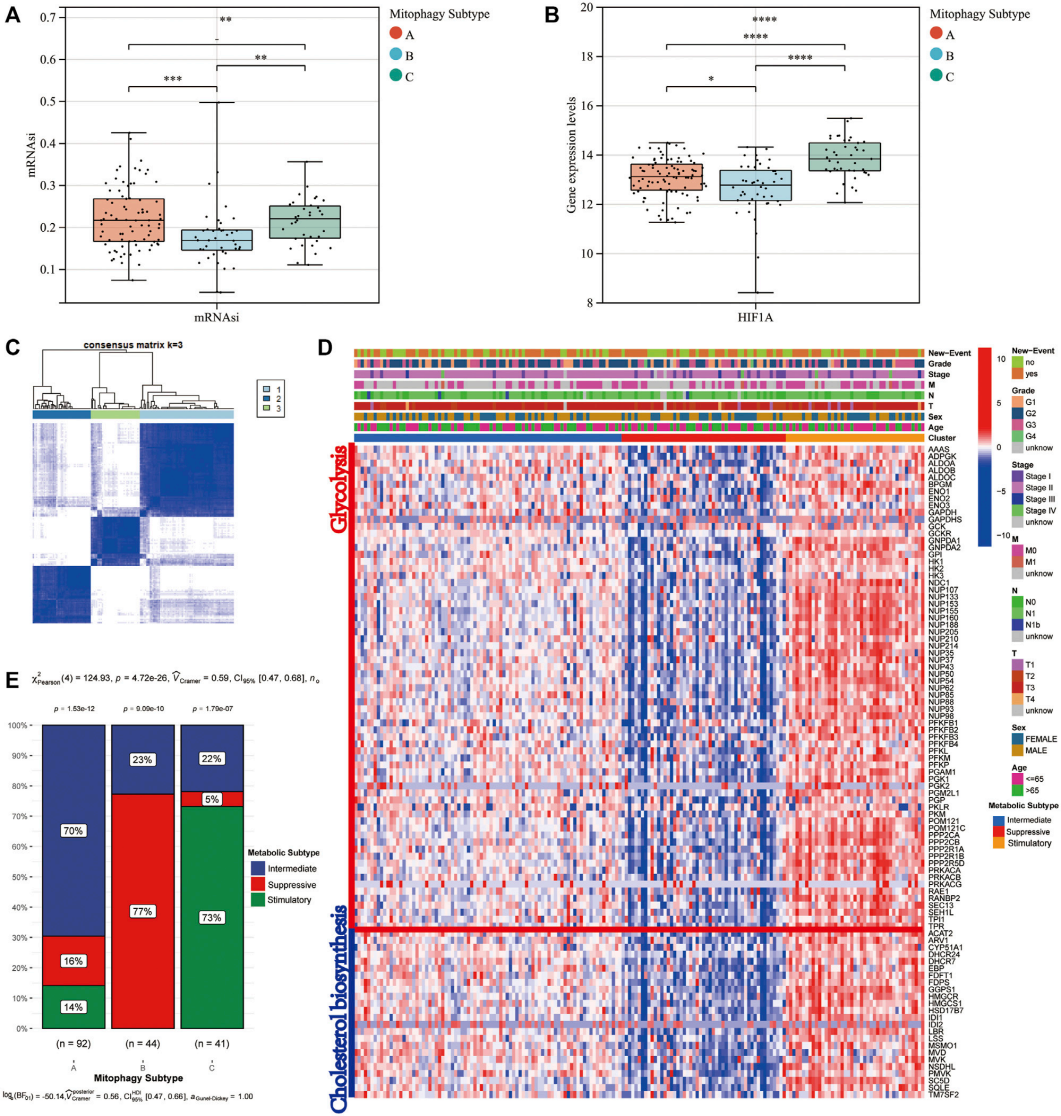
Difference in mRNAsi level **(A)** and HIF1A **(B)** gene expression level according to mitophagy subtypes. Identify different metabolic subtype. **(C)** Consensus matrices of the TCGA-PAAD cohort for *k* = 3. **(D)** Heat map comparing expression levels of cholesterol biosynthesis and glycolysis related genes among the 3 mitophagy subtypes. **(E)** The proportion of metabolic subtypes in the three mitophagy subtypes, *p* values were from the chi-squared test (Intermediate subtype, blue; suppressive subtype, red; stimulatory subtype, green).

The mRNA expression-based stemness index (mRNAsi) of all patients in the TCGA-PAAD cohort was downloaded from the study by Malta et al. mRNAsi can be used to evaluate the dedifferentiation potential of tumour cells; therefore, it is considered as a marker of CSCs. The median mRNAsi score was the lowest in cluster B, with no significant difference in mRNAsi scores between clusters A and C (Figure 4A). Moreover, HIF1α, a hypoxia marker, expression levels were evaluated in the three mitophagy subtypes, revealing a significant increase in cluster C (Figure 4B).

We performed unsupervised hierarchical clustering based on ninety-seven major regulators of cholesterol biosynthesis and glycolysis. Then we identified three metabolic subtypes in the TCGA-PAAD cohort (intermediate, suppressive and stimulatory; Figures 4C,D). The gene expression patterns for ninety-seven major regulators of cholesterol biosynthesis and glycolysis in the TCGA-PAAD cohort were presented in Figure 4D. Cluster A, the intermediate mitophagy subtype, demonstrated a significantly high level in the intermediate metabolic subtype (70%; Chi-square test *p-*value < 0.0001). Conversely, a significantly greater proportion of stimulatory metabolic subtype was observed in cluster C (Chi-square test *p*-value < 0.0001). A large proportion of the suppressive subtype was observed in cluster B; however, the stimulatory metabolic subtype was not observed in cluster B (Chi-square test *p*-value < 0.0001; Figure 4E).

### Construction of weighted Co-expression network construction and the identification of key modules

**FIGURE 5:**
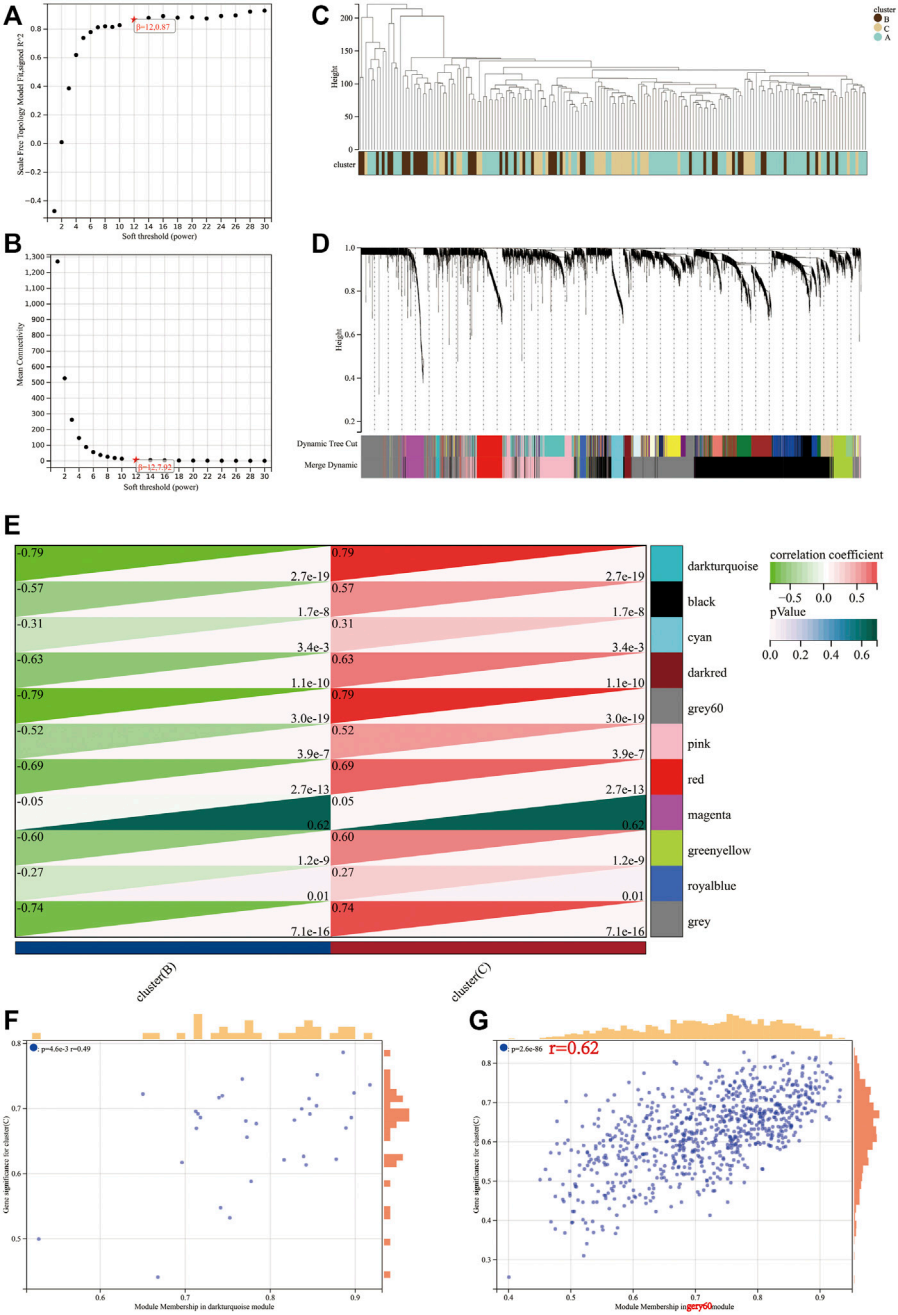
Determination of soft-threshold power in the WGCNA. **(A)** Analysis of the scale-free index for various soft-threshold powers (*β*). **(B)** Analysis of the mean connectivity for various soft-threshold powers. **(C)** Clustering dendrogram of 176 samples. Identification of modules closely associated with mitophagy subtypes. **(D)** Dendrogram of all differentially expressed genes clustered based on the measurement of dissimilarity (1-TOM). The color band shows the results obtained from the automatic single-block analysis. **(E)** Heatmap of the correlation between the module eigengenes and mitophagy subtype of PAAD. A scatterplot of gene significance (GS) for high mitophagy subtype (cluster C) versus module membership (MM) in the darkturquoise module **(F)** and gery60 module **(G)**.

The transcriptional profiles of the 9,221 genes in the 178 tumour samples of PAAD were utilised to establish the co-expression module using the WGCNA R package. Power value is a major parameter affecting the independence and average connectivity degree. Soft power 12 was used as the soft threshold to construct a weighted adjacency matrix (Figures 5A,B). A total of 4,515 genes were assigned to one of the 11 co-expression modules including the grey module in the TCGA-PAAD cohort. The results of the cluster analysis on PAAD samples are demonstrated in Figure 5C. These co-expression modules are represented using different colours (Figure 5D). The affiliation of genes to modules is recorded in Supplementary Table S3.

The module–trait correlations heatmap indicate that the dark turquoise and grey60 modules were tightly associated with the mitophagy subtypes (correlation coefficient = 0.79, *p-*value < 0.0001; Figure 5E). The scatter plot of GS versus module membership (MM) for the dark turquoise and grey60 modules are depicted in Figures 5F,G. Correlation analysis suggested a larger coefficient and smaller *p*-value for the grey60 module than the dark turquoise module. Therefore, grey60 was identified as a characteristic module of the mitophagy subtype.

A total of 800 unique genes comprised the grey60 module. Based on the criteria of |MM| > 0.8 and |GS| > 0.1, 218 genes in the grey60 module were screened out as hub genes (Supplementary Table S4).

**FIGURE 6:**
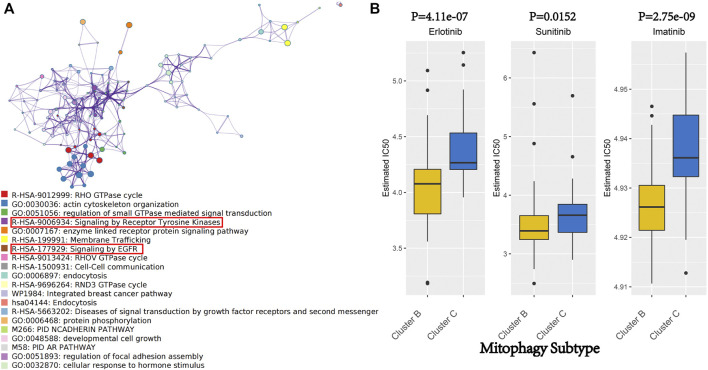
**(A)** Network of enriched terms of hub genes of gery60 module by Metascape. Colored by cluster ID, where nodes that share the same cluster ID are typically close to each other. **(B)** We evaluated the IC 50 of Erlotinib, Sunitinib and Imatinib, between cluster B and cluster C by performing the R package “pRRophetic.”

Metascape web tool carried out the pathway enrichment analysis based on the 218 hub genes and the top 20 clusters with their representative enriched terms (one per cluster) are presented in detail in Supplementary Table S5 (Figure 6A). The enriched terms included GTPase cycles (R-HSA-9012999, GO:0051056, R-HSA-9013424, R-HSA-9696264), membrane protein and functions (GO:0007167, R-HSA-1500931, GO:0006897, hsa04144) and tyrosine kinases and EGFR signaling pathways (R-HSA-9006934 and R-HSA-177929).

### Therapeutic potential of mitophagy subtypes in pancreatic adenocarcino

Erlotinib and Sunitinib are anticancer drugs approved by the United States Food and Drug Administration for pancreatic cancer treatment. Erlotinib is a novel, oral, highly selective tyrosine kinase inhibitor (TKI) of the EGFR, whereas Sunitinib is an oral, multi-targeted TKI with low molecular weight. As a rationally designed oral signal transduction inhibitor, Imatinib can specifically target several tyrosine kinases. Imatinib was approved for the treatment of chronic myeloid leukaemia and malignant gastrointestinal stromal tumours. Metascape analysis indicated that the hub genes of the grey60 module were enriched in the pathways involved in the anticancer activity of Erlotinib, Sunitinib and Imatinib (R-HSA-9006934, R-HSA-177929; Figure 6A). Therefore, these three drugs were focused on as patients from different mitophagy subtypes may have varied responses to these drugs. The IC50 was evaluated using the R package “pRRophetic.” Moreover, the treatment response to Erlotinib, Sunitinib and Imatinib in the TCGA-PAAD cohort was predicted, wherein low mitophagy (cluster B) subtypes were more sensitive to Erlotinib, Sunitinib and Imatinib (*p*-value < 0.05; Figure 6B) than clusters A and C.

### Identification of candidate MSGSs using machine learning techniques

**FIGURE 7:**
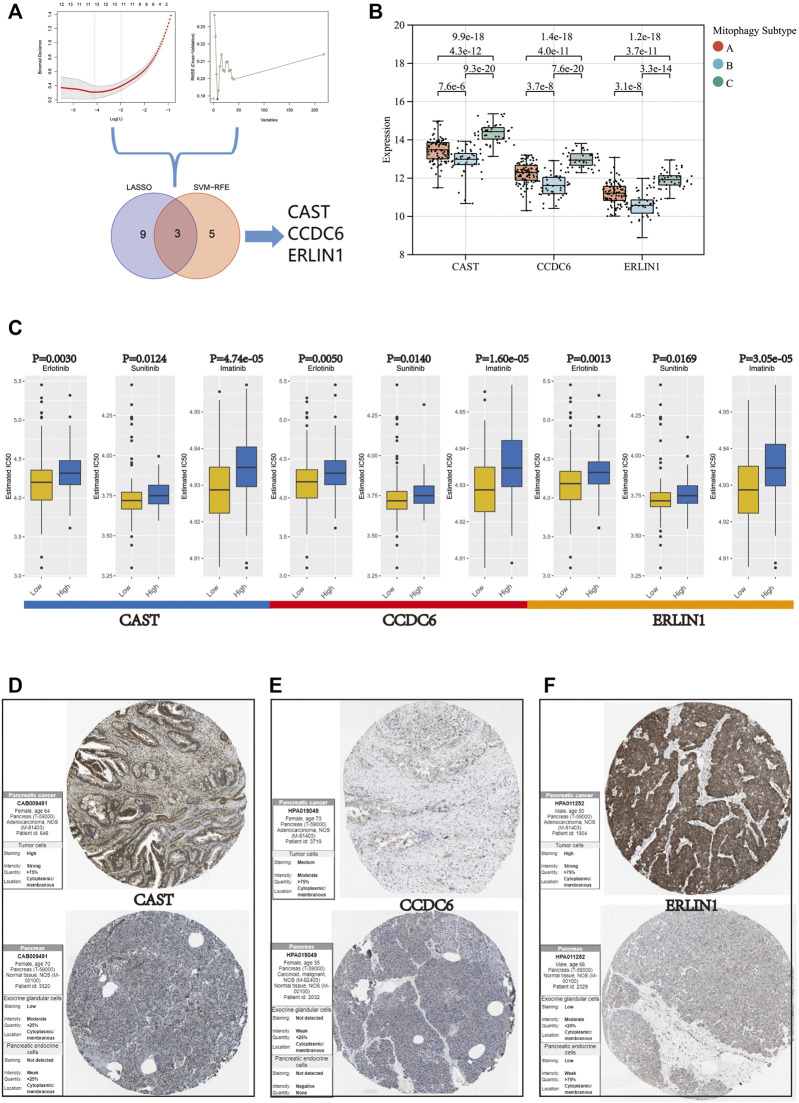
**(A)** Flow chart of the screening procedure. **(B)** Variation in gene expression levels of CAST, CCDC6, and ERLIN1 across different mitophagy subtypes (*p*-values were calculated by the Kruskal Wallis test). **(C)** Differences in IC 50 of Erlotinib, Sunitinib and Imatinib evaluated by R package “pRRophetic” between the high and low groups of CAST, CCDC6, and ERLIN1. Immunohistochemistry analysis of CAST **(D)**, CCDC6 **(E)**, and ERLIN **(F)** expression in HPA database.

Two algorithms were used to screen for potential biomarkers between the low (cluster B) and high (cluster C) mitophagy subtypes in the TCGA-PAAD cohort. The 218 hub genes in the grey60 module were used to dimensionality reduction analysis by the LASSO regression algorithm. A total of 12 variables were obtained as diagnostic biomarkers (Figure 7A). Eight features among the 218 hub genes in the grey60 module were also identified through the SVM-RFE algorithm (Figure 7A). Finally, the three overlapping features (*CAST, CCDC6, ERLIN1*) between the two algorithms were considered as the candidate MSGSs. The three MSGSs were assessed using the GEPIA database and further evaluated on their similarity to the primary characteristics of low or high mitophagy subtypes (Supplementary Figure S4). Gene expression levels of *CAST, CCDC6* and *ERLIN1* was upregulated in PAAD tissues compared with those of the normal tissues. *CAST* and *CCDC6* high expression indicated a poor prognosis of PAAD. Additionally, CAST high expression was significantly associated with worse disease-free survival (*p-*value = 0.033). Kruskal–Wallis test indicated that *CAST, CCDC6,* and *ERLIN1* expression levels were the highest in the high mitophagy (cluster C) subtype and lowest in the low mitophagy (cluster C) subtype (Figure 7B).

Furthermore, the mRNA expression of *CAST, CCDC6* and *ERLIN1* could potentially predict the tumour’s sensitivity to Erlotinib, Sunitinib and Imatinib. Samples in the TCGA-PAAD cohort were segregated into two groups according to the median value for *CAST, CCDC6,* and *ERLIN1*. Moreover, *CAST, CCDC6* and *ERLIN1* low expression are suggestive of a lower IC50, indicating their potential as novel indicators for the drug susceptibility of Erlotinib, Sunitinib and Imatinib (Figure 7C).

Based on the HPA database, immunohistochemistry suggested that CAST, CCDC6, and ERLIN1 protein expression was lower in normal pancreatic tissues but higher in pancreatic cancer tissues (Figures 7D–F).

### Relationship between the tumour microenvironment and MSGSs

**FIGURE 8:**
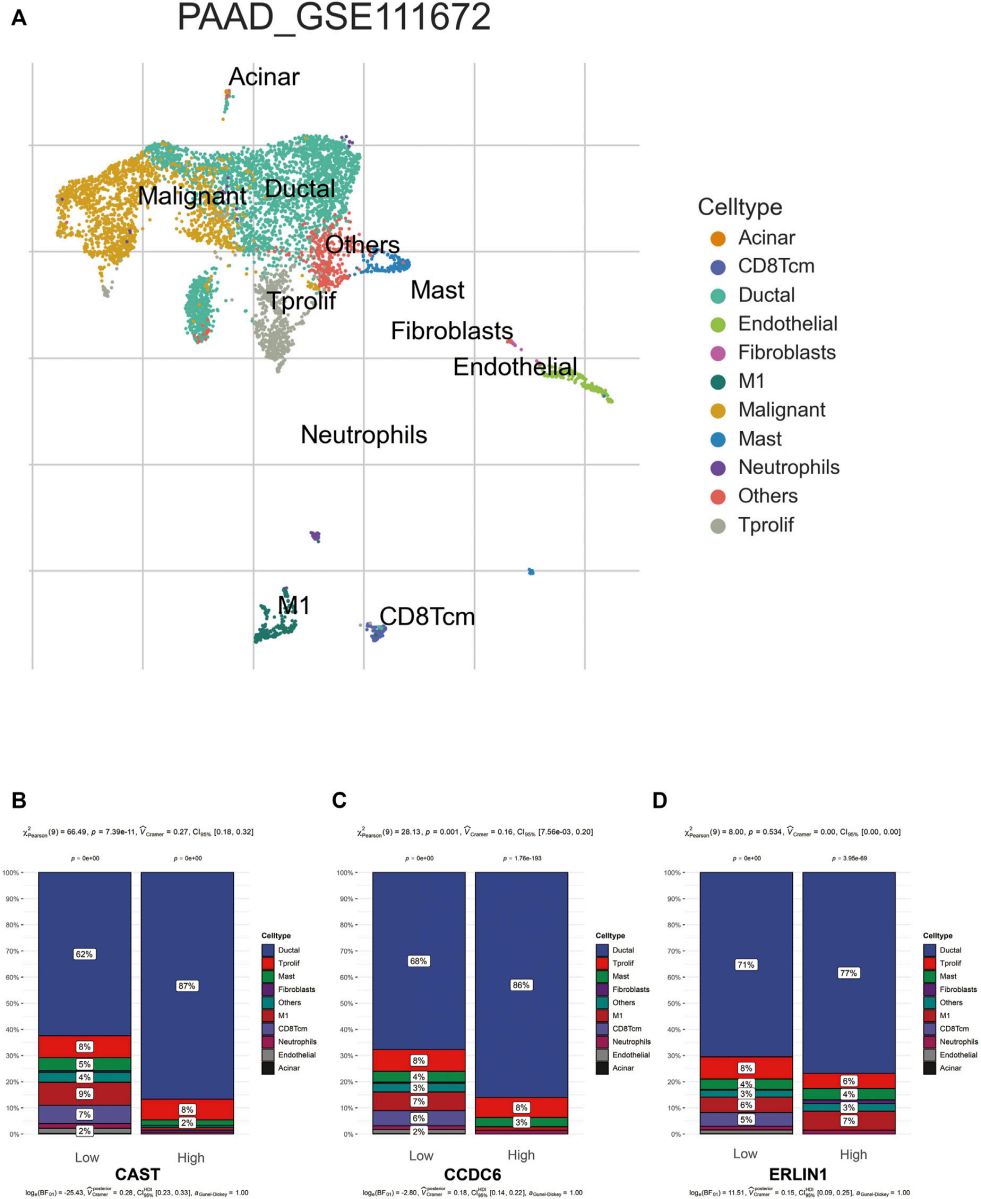
**(A)** TISCH provides detailed cell type annotation at the single cell level of GSE111672. The proportion of different kinds of non-cancerous cells infiltrated in the high and low groups of CAST **(B)**, CCDC6 **(C),** and ERLIN **(D)**. *p* values were from the chi-squared test.

TISCH was used to perform quality control, clustering and cell-type annotation for the single-cell RNA-sequencing dataset GSE111672. A total of 11 cell types were identified in GSE111672 (Figure 8A). Samples in GSE111672 were separated into two groups according to high or low CAST, CCDC6 and ERLIN1 expressions. Furthermore, the infiltration pattern of non-cancerous cells between the three groups was compared. Ductal cells were significantly higher in the high CAST expression group than that with low CAST. Notably, a higher percentage of men was observed in the alcohol group. Furthermore, high percentages of M1 and central memory CD8^+^T (CD8Tcm) cells were observed in the low CAST expression group (Figure 8B). A similar infiltration pattern of non-cancerous cells was observed in the high/low CCDC6 expression groups (Figure 8C). Additionally, CD8Tcm cells were significantly higher in the low ERLIN1 expression group than that with highERLIN1 expression (Figure 8D).

**FIGURE 9:**
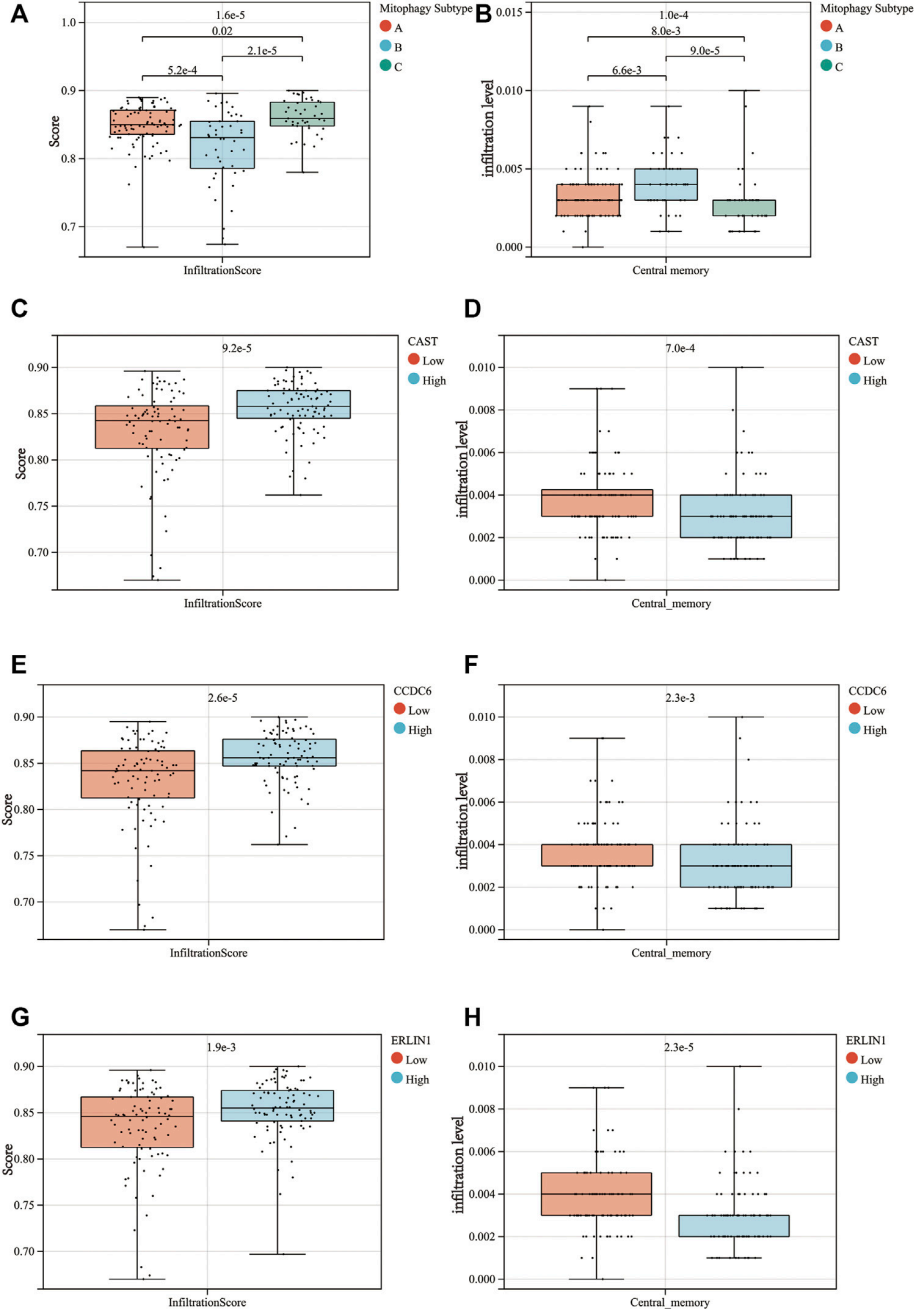
Differences in the infiltrationscore **(A)** and the central memory infiltration **(B)** among the 3 mitophagy subtypes (*p*-values were calculated by the Kruskal Wallis test). Differences in the infiltrationscore and the central memory infiltration between the high and low groups of CAST **(C,D)**, CCDC6 **(E,F),** and ERLIN1 **(G,H)** (*p*-values were calculated by the Wilcoxon test).

For further validation of the results, the degree of immune cell infiltration in the TCGA-PAAD cohort was assessed using the ImmuCellAI web tool. The results indicated that the high mitophagy (cluster C) subtype tumours had high immune cell infiltration levels, whereas the low mitophagy (cluster B) subtype had low immune cell infiltration levels (Figure 9A). The further subdivision of central memory T cells could not be achieved using the ImmuCellAI, thus, only the infiltration of central memory T cells was evaluated. Among the three mitophagy groups, the infiltration of central memory T cell was highest in the low mitophagy (cluster B) subtype and lowest in the high mitophagy (cluster C) subtype (Figure 9B). The high CAST expression group was characterised by high immune cell infiltration levels and low central memory T cell infiltration levels (Figures 9C,D). The same characteristics were also observed in the high CCDC6 and high ERLIN1 groups (Figures 9E–H).

### Novel indicators for screening chemosensitive patients

**FIGURE 10:**
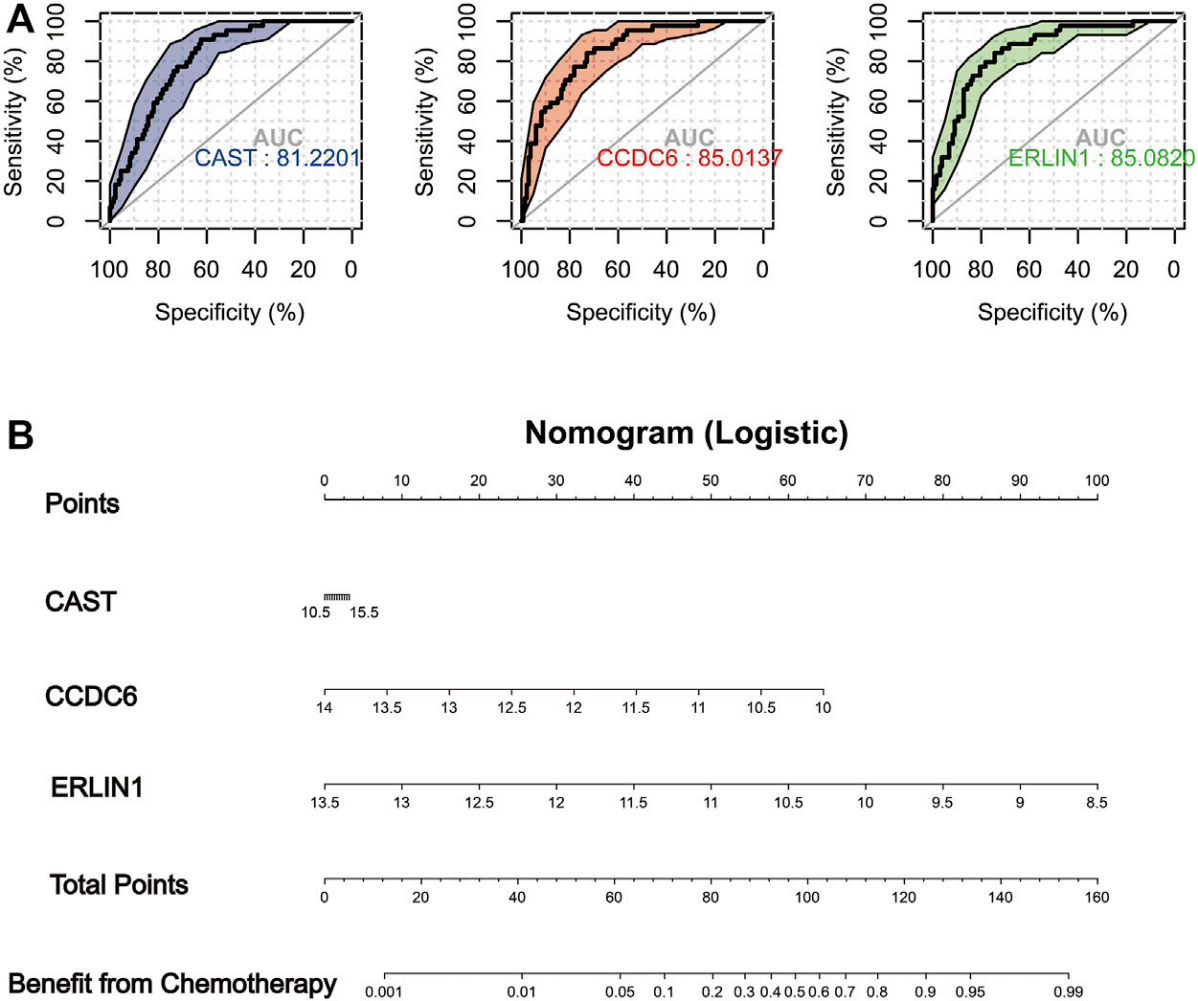
**(A)** In TCGA-PAAD cohort, ROC analysis evaluating diagnostic accuracy of CAST, CCDC6 and ERLIN1 in low mitophagy subtype (cluster B). **(B)** Construction of a nomogram based on the gene expression of CAST, CCDC6 and ERLIN1 to identify PAAD patients can more benefit from chemotherapy (cluster B).

The IC50 of Erlotinib, Sunitinib and Imatinib was lower in the low mitophagy subtype (Cluster B); therefore, patients in cluster B were indicated as those who might benefit from chemotherapy. Other clusters (clusters A and C) were considered as doubtful or unsuitable for the chemotherapy of Erlotinib, Sunitinib or Imatinib. Therefore, it is crucial to accurately triage and identify low mitophagy subtype (cluster B) PAAD cases early in clinical treatment. Subsequently, based on ROC curve analysis, the potential of the three MSGSs as diagnostic biomarkers for the low mitophagy subtype were assessed (Figure 10A). ROC curve analyses indicated the following diagnostic accuracies : *CAST* (AUC = 0.812), *CCDC6* (AUC = 0.850) and *ERLIN1* (AUC = 0.851). Moreover, a nomogram was established based on the gene expression of *CAST, CCDC6* and *ERLIN1* (Figure 10B). This nomogram was used to assess the probability of being low mitophagy subtype in PAAD patients. The generation of nomogram provided a tool for estimating probability of benefiting from chemotherapy.

## Discussion

Mitophagy contributes significantly to the tumour microenvironment, tumour metabolism and tumour prognosis. Previous studies have mainly focused on the associations between individual regulators and cancer phenotypes, however, the distinct subtypes based on the overall characterization of various mitophagy regulators remain insufficiently identified (Zhou et al., 2011) (Sliter et al., 2018) (Cho et al., 2020). Exploring the distinct mitophagy alteration patterns in PAAD could aid in understanding the occurrence and progression of PAAD, inspiring novel and innovative strategies for its treatment and prognosis.

This study assessed the expression levels of various mitophagy regulators in normal and PAAD tissues, revealing that a majority of them were significantly upregulated in the tumour samples, except ULK1. As a critical initiator of mitophagy, ULK1 was reported to be downregulated in various solid tumours (Li et al., 2021) (Deng et al., 2021). Further exploration indicated that CNV alterations could be largely responsible for the perturbations of some mitophagy regulators, particularly ULK1, CSNK2B and SQSTM1 expressions. Thus, the alterations of CNV in mitophagy regulators could be a potential underlying cause of mitophagy heterogeneity. The diagnostic capacity of the mitophagy regulators in PAAD was evaluated using the dimensionality reduction of gene expression patterns of mitophagy regulators, which revealed that UMAP1 and UMAP2 could be potential diagnostic biomarkers for PAAD (Marquardt et al., 2021). Furthermore, three independent datasets were used to validate these results. Some datasets indicated that UMAP1 and UMAP2 were more valuable for PAAD diagnosis than several traditional tumour biomarkers (Chang and Kundranda, 2017).

Mitophagy alteration patterns were explored, which revealed three distinct molecular subtypes based on the integrated role of various mitophagy regulators in PAAD. Significant differences in mitophagy accumulation among the subtypes were observed, with patients in the high mitophagy subtype (cluster C ) having the worst prognosis. Stage, age, gender and new events showed no significant difference between the three subtypes, suggesting that the poor prognosis in cluster C was not driven by the clinical baseline. To further explain these findings, the level of mRNAsi and HIF1A gene expression were evaluated (Zhang et al., 2020) (de Heer and Harris, 2020). A lower mRNAsi level appeared primarily in the low mitophagy subtype (cluster B ) than that in the high mitophagy subtype (cluster C). Previous studies have described mRNAsi as the indices of CSC characteristics that apply to different tumour types. The presence of CSCs is a leading cause of tumour recurrence, drug resistance and poor prognosis (Nagler et al., 2011). Therefore, the better prognosis of cluster B than cluster C could be attributed to the low level of CSCs. Additionally, the high mitophagy subtype (cluster C ) shows high levels of *HIF1A* expression, indicating that the tumour in cluster C has a strong hypoxic environment. Hypoxia has been reported to be a tumour microenvironmental hallmark, indicating poor prognosis in most solid tumours (Jing et al., 2019). Liu et al. (2012) reported that the hypoxia-induced dephosphorylation of *FUNDC1* enhanced its interaction with LC3 for selective mitophagy. Furthermore, lactic acid produced in a hypoxic environment lowers the pH of the tumour microenvironment, which significantly weakens the function of normal immune cells, such as T cells and tumour-infiltrating lymphocytes (Wang et al., 2020). Additionally, the synthesis of hyaluronic acid by tumour-associated fibroblasts in a high lactate environment promotes the growth and activity of cancer cells at certain concentrations (Stern, 2008) (Walenta and Mueller-Klieser, 2004). This phenomenon could explain the worst prognosis of the high mitophagy subtype compared to other subtypes to a certain extent.

Several studies have reported the impact of mitophagy on tumour metabolic reprogramming. PAAD includes not only *KRAS* and *TP53* mutations but also high hypoxia levels, which induces the glycolysis pathway in cancer (Kamisawa et al., 2016) (Yang et al., 2020) (Lin et al., 2018) (Nagdas et al., 2019). The conversion of the glycolytic metabolite pyruvate to lactic acid or its transport to the mitochondria *via* the mitochondrial pyruvate complex attenuates the glycolytic tumour-promoting effects (Semenza, 2011). Pyruvate, a metabolic intermediate of the tricarboxylic cycle, provides citric acid precursors for cholesterol and free fatty acid biosynthesis (Park et al., 2015). High levels of cholesterol are required to fulfil the needs of membrane biogenesis during tumour cell proliferation (Huang et al., 2020). Therefore, cholesterol metabolism generally promotes cancer cell proliferation, migration and invasion (Goossens et al., 2019). Furthermore, on exploring the tumour metabolic patterns based on glycolysis and cholesterol biogenesis, the metabolic heterogeneity between the three mitophagy subtypes was found to be crucial. The vast majority of patients in the high mitophagy subtype (cluster C) exhibited high levels of glycolysis and cholesterol biogenesis. Additionally, the inhibition of glycolysis and cholesterol biogenesis was observed in the low mitophagy subtype (cluster B). The highly hypoxic microenvironment in cluster C could be responsible for the more active glycolysis than clusters A and B. Roca-Agujetas et al. suggested that high intracellular cholesterol levels upregulated the mitochondrial PINK1 accumulation to induce mitophagosomes formation. Although this phenomenon was documented in cholesterol-enriched SH-SY5Y cells and cultured primary neurons, it partially concurs with the findings of this study. Both glycolysis and cholesterol biogenesis have been reported as risk factors for poor prognosis in various human tumours (Nelson, 2018) (Munir et al., 2018) (Vander Heiden and Thompson, 2009). Therefore, the metabolic heterogeneity contributes to the difference in prognosis between the three mitophagy subtypes.

WGCNA algorithm was used to identify mitophagy subtype-related gene co-expression modules. Hub genes in the module obtained were enriched in signaling by receptor tyrosine kinases and EGFR. The major mechanism underlying the anticancer effects of Erlotinib, Sunitinib and Imatinib is the induction of receptor tyrosine kinases and EGFR signaling (Abdelgalil et al., 2020) (Ferrari et al., 2019) (Waller, 2018). Consistently, a significant difference in drug sensitivity was observed between the high (cluster C) and low mitophagy (cluster B) subtypes. Reddy et al. identified anti-mitophagy as a kinase-independent function of EGFR and revealed a new function of the mTORC2/Akt axis in promoting mitophagy in tumour cells (Katreddy et al., 2018). A study by Lyons et al. reported that MCF-7 cells with acquired resistance to an IGF-1 receptor TKI reduced mitochondrial biogenesis. Notably, the cells revealed mitochondrial dysfunction, which was indicated by the presence of reactive oxygen species expression, reduced expression of the mitophagy mediators BNIP3 and BNIP3L and impaired mitophagy (Lyons et al., 2017).

Furthermore, a machine learning pipeline was utilised to identify the signature of the high mitophagy subtype (cluster C), revealing three MSGSs (*CAST, CCDC6* and *ERLIN1*). These MSGSs have the potential to be diagnostic and tumour subtyping biomarkers in PAAD. Additionally, they could be used as, novel indicators of chemotherapeutic drug sensitivity to Erlotinib, Sunitinib and Imatinib.

As an endogenous calpain, CAST is an important participant in proteolysis of amyloid precursor protein and multiple membrane fusion events. Membrane fusion is fundamental to the degradation process that delivers cytoplasmic material to lysosomes *via* autophagosomes. Thus, membrane fusion mediated by CAST could be one of the principal mechanisms for the degradation of the mitochondria during the mitophagy (Liu and Zhong, 2021). CCDC6 is involved in triggering a DNA damage checkpoint response and maintaining genomic stability. CCDC6 mutation decreases the apoptotic response in response to DNA damage, leading to the development of radio- and chemoresistance. In addition, the study of Xiuli et al. demonstrated obvious mitophagy increases with the severity of DNA damage in primary fibroblasts, murine neurons and *Caenorhabditis elegans* neurons (Cerrato et al., 2018). Therefore, CCDC6 appears to influence mitophagy the by regulating the DNA damage repair. As a important communication subdomains between endoplasmic reticulum (ER) and mitochondria, mitochondria-associated membranes (MAMs) are the primary site of interaction between ERLIN1 and AMBRA1. A gene knock-out experiment confirmed that ERLIN1 interacts with AMBRA1 in MAM raft-like microdomains and results in the formation of autophagosomes (Manganelli et al., 2021). As described above, the obtained MSGSs are reasonable and are closely related to mitophagy.

Increasing evidence reveals mitophagy as a crucial factor in the maintenance of the immune system, owing to the elimination of dysfunctional mitochondria (Song et al., 2020). Mitochondrial antigen presentation and immune cell homeostasis can be directly regulated by inflammatory cytokine secretion, which is downregulated by mitophagy (Zhong et al., 2016) (Patoli et al., 2020). Furthermore, on exploring the infiltration pattern of immune cells in PAAD, immune cell infiltration was found to be upregulated in the group with higher MSGS expression. Single cell sequencing analysis indicate that CD8Tcm infiltrate is significantly different between the low and high MSGS groups, with similar results observed in the ImmuCellAI analysis. Previous studies have revealed that memory T cells, including stem cell memory (Tscm) T cells and central memory (Tcm) T cells, exhibit superior persistence and antitumour immunity compared to effector memory T (Tem) cells and effector T (Teff) cells. Moreover, the Tcm/Teff ratio has been considered as an evolving biomarker for immunotherapy response (Liu et al., 2020). These results indicate the clinical potential of MSGSs in screening out immunotherapy resistant populations in PAAD.

Additionally, the proportion of M1 macrophages in the high CAST and CCDC6 groups was significantly higher than that in the low CAST and CCDC6 groups. However, a significant difference was observed in the M1 macrophage proportion between the high and low ERLIN1 groups. Generally, M2 (repair-type) macrophages are predominate in human tumours and secrete growth-promoting molecules that stimulate tumour proliferation. However, converting M2 macrophages to M1 (kill-type) slows down proliferation (Charles and Harris, 2016). Although *CAST, CCDC6* and *ERLIN1* are gene signatures of the high mitophagy subtype (cluster C), Kaplan-Meier analysis suggests that *CAST* and *CCDC6* are risk factors for the prognosis of patients with PAAD, while *ERLIN1* does not affect the prognosis (Supplementary Figure S4). The infiltration and differentiation of macrophage could be responsible for the differences in prognosis (Hwang et al., 2020).

Patients in the low mitophagy subtype (cluster B) had a better prognosis and higher chemotherapy sensitivity than the other subtypes. The identification of patients in cluster B could aid in individualising treatment regimens. ROC analyses revealed that MSGSs are a better screening factor for cluster B patients. Additionally, three MSGSs were incorporated to construct a nomogram, which has potential clinical applications.

This study sheds new light on potential strategies that could be used in personalising treatment regimens for patients with PAAD. However, this study has certain limitations. The current study focused on bioinformatic analyses and lacks experimental and clinical validation. Additionally, this research was retrospective rather than prospective (Talari and Goyal., 2020). However, the results are based on multiple independent cohorts; therefore, it remains credible and acceptable. Hence, further exploration of molecular mechanisms and prospective clinical trials are warranted to validate the current results.

## Conclusion

Using unsupervised clustering based on mitophagy regulators, three distinct mitophagy subtypes with different metabolism patterns and prognoses were obtained in PAAD. The high mitophagy subtype had the poorest prognosis and highest level of glycolysis and cholesterol biosynthesis. The low mitophagy subtype displays higher sensitivity to Erlotinib, Sunitinib and Imatinib. Then, a new mitophagy-associated risk score system were established to provide a potential prognostic predictor for PAAD. Additionally, using WGCNA and machine learning, three diagnostic gene signatures (CAST, CCDC6, and ERLIN1) for the high level mitophagy subtype were obtained, which were closely associated with tumour immune microenvironment and chemotherapy sensitivity in PAAD. Futhermore, a nomogram was conducted based on the gene expression of CAST, CCDC6, and ERLIN1. The generation of nomogram provided a tool for estimating the probability of benefiting from chemotherapy. This study establishes the foundation for further investigation of mitophagy in the tumorigenesis and tumour development of PAAD. Furthermore, it contributes to the development of personalised clinical management and treatment regimens of PAAD.

## Data Availability

Publicly available datasets were analyzed in this study. This data can be found in The Cancer Genome Atlas (TCGA) database (https://portal.gdc.cancer.gov/), Gene Expression Omnibus (GEO) database (https://www.ncbi.nlm.nih.gov/geo/) and International Cancer Genome Consortium (ICGC) data portal (https://icgc.org/).

## Supplementary material

Supplementary Figure S1 Mechanisms and regulation of mitophagy in mammalian cells.

Supplementary Figure S2
**(A)** Prognostic mitophagy regulators determined by the univariate Cox regression analyses. **(B)** Risk score prognostic model construction by LASSO logistic regression analysis. **(C)** The patients were classified into the high risk or low-risk group according to the median riskscore calculated by the prognostic model. The Kaplan–Meier curve indicated a high level of risk score, suggesting that patients have a poor prognosis (Log rank *p*-value = 0.0035). **(D)** ROC curve of prognostic model predicting 5-year OS.

Supplementary Figure S3
**(A)** Differences in expression of mitophagy regulators in different mitophagy subtypes. Identify different mitophagy subtypes in PACA-AU cohort. **(B)** Consensus matrices of the PACA-AU cohort for *k* = 3. **(C)** K-M analyses of OS for different mitophagy subtypes in PACA-AU cohort. **(D)** Heat map comparing expression levels of mitophagy regulators among the 3 mitophagy subtypes.

Supplementary Figure S4 With the GEPIA database, we compared the expression levels of CAST, CCDC6 and ERLIN1 in PAAD tumors and normal tissues (red color represents tumor tissue, and black color represents normal tissue; red asterisk represents a *p*-value ≤ 0.01). Kaplan-Meier curves (OS and DFS) for expression of CAST , CCDC6, and ERLIN1 (*p*-values were obtained from Log rank test).

Supplementary Table S1 Signaling pathway associated with mitophagy,cholesterol biosynthesis and glycolysis.

Supplementary Table S2 The results of Univariate Cox regression analysis in TCGA-PAAD cohort.

Supplementary Table S3 The affiliation of genes to modules.

Supplementary Table S4 Hub genes in grey60 module.

Supplementary Table S5 Enrichment results of hub genes by Metascape.
