# Supplementary figures and images for: Mitophagy-mediated molecular subtypes depict the hallmarks of the tumour metabolism and guide precision chemotherapy in pancreatic adenocarcinoma
Source: Front Cell Dev Biol. 2022 Jul 22;10:901207. doi: 10.3389/fcell.2022.901207 (PMC9353335; doi:10.3389/fcell.2022.901207)

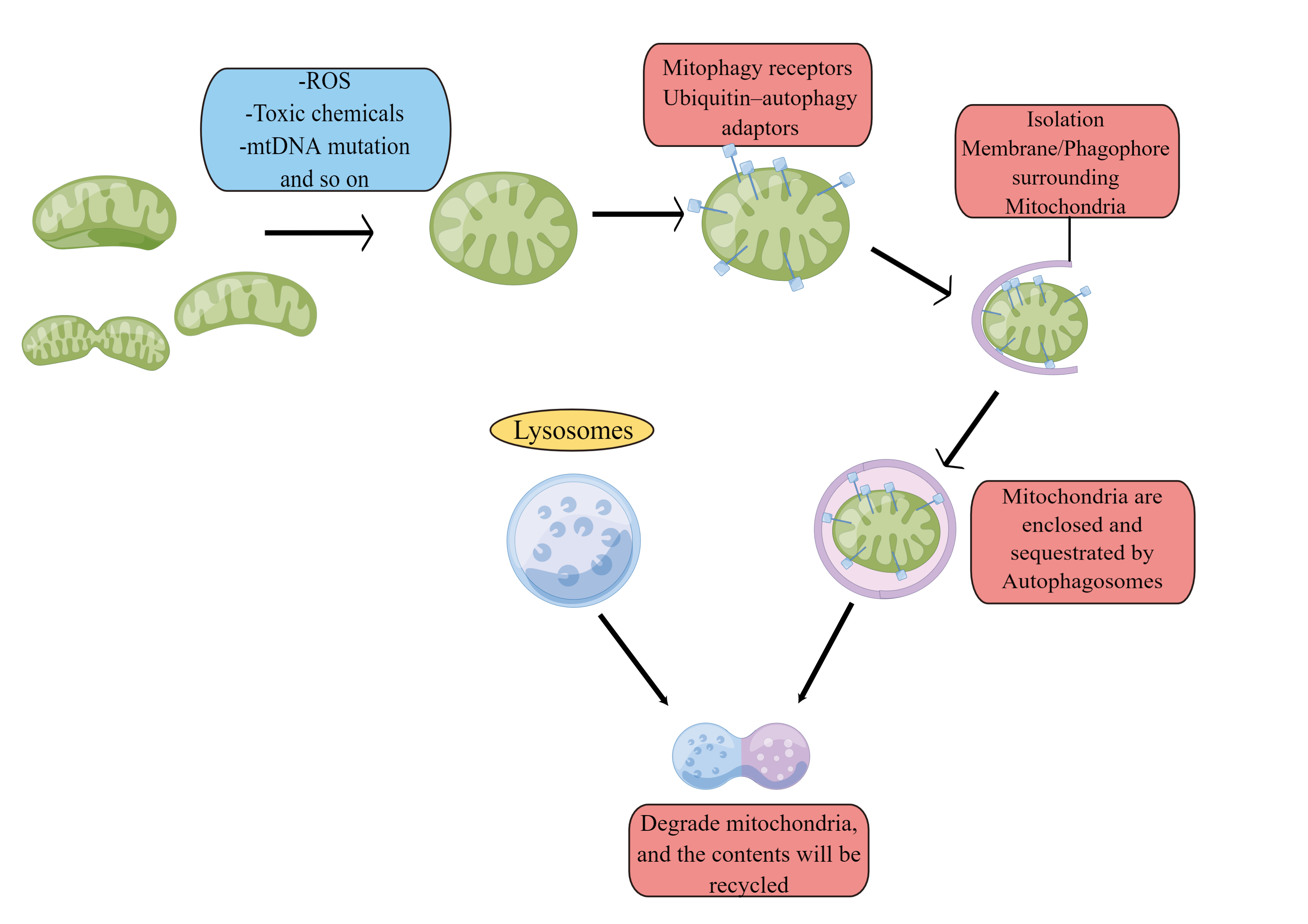

Supplement: Supplementary file 2 [file Image1.jpeg]
